# Staged revascularization vs. culprit-only percutaneous coronary intervention for multivessel disease in elderly patients with ST-segment elevation myocardial infarction

**DOI:** 10.3389/fcvm.2022.943323

**Published:** 2022-09-09

**Authors:** Jiachun Lang, Chen Wang, Le Wang, Jingxia Zhang, Yuecheng Hu, Huajun Sun, Hongliang Cong, Yin Liu

**Affiliations:** ^1^Clinical School of Thoracic, Tianjin Medical University, Tianjin, China; ^2^Department of Cardiology, Tianjin Chest Hospital, Tianjin, China; ^3^School of Public Health, Tianjin Medical University, Tianjin, China; ^4^Tianjin Basic Public Health Service Quality Control Center, Tianjin, China

**Keywords:** elderly, ST-segment elevation myocardial infarction, multivessel disease, percutaneous coronary intervention, staged revascularization

## Abstract

**Backgroundand objective:**

Studies have highlighted the significant role of staged percutaneous coronary intervention (PCI) for a multivessel disease (MVD) among patients with ST-elevation myocardial infarction (STEMI). However, the relative benefit of staged vs. culprit-only PCI for MVD in elderly patients with STEMI remains undetermined. Thus, the present study compared the clinical outcomes of staged and culprit-only PCI in this cohort.

**Methods:**

From January 2014 to September 2019, 617 patients aged ≥65 years with STEMI and MVD who underwent primary PCI of the culprit vessels within 12 h of symptom onset were enrolled. They were then categorized into the staged and culprit-only PCI groups according to intervention strategy. Propensity score matching (PSM) was conducted to adjust for confounding factors between groups. The primary end point was major adverse cardiac and cerebrovascular events (MACCE), a composite of all-cause death, cardiac death, recurrent myocardial infarction (MI), stroke, and ischemia-driven revascularization.

**Results:**

During a mean follow-up of 56 months, 209 patients experienced MACCE and 119 died. Staged revascularization was associated with a lower risk of MACCE, all-cause death, and cardiac death than culprit-only PCI in both overall patients and the PSM cohorts. In contrast, there was no significant difference in stroke or ischemia-driven revascularization. Moreover, on multivariate Cox regression analysis, staged PCI was a significant predictor of a lower incidence of MACCE and all-cause death.

**Conclusion:**

In elderly patients with STEMI and MVD, staged PCI is superior to culprit-only PCI.

## Introduction

An ST-segment elevation myocardial infarction (STEMI) is the most threatening type of coronary heart disease. Primary percutaneous coronary intervention (PCI) within 12 h of symptom onset is the best revascularization strategy for patients with STEMI (1). Approximately 40–65% of patients with STEMI have multivessel disease (MVD) (2–5). A lower incidence of reperfusion success, a lower left ventricular ejection fraction (LVEF), and a higher mortality rate were reported for STEMI patients with MVD vs. single-vessel disease. There are three intervention strategies for MVD in patients with STEMI: (1) culprit-only vessel PCI during the index procedure; (2) culprit vessel revascularization during primary PCI with non-culprit vessels staged revascularized after primary PCI; and (3) multivessel revascularization during primary PCI. Previous studies (6–9) confirmed that culprit-only PCI is superior to multivessel revascularization during primary PCI. Recent studies (10) elucidated that for MVD in patients with STEMI, staged PCI could reduce mortality more than culprit-only PCI. Moreover, recent meta-analyses (11) demonstrated that staged PCI has a better effect than multivessel primary PCI. Therefore, the latest guidelines (1) upgraded the recommendation of staged PCI for MVD over culprit-only PCI. Moreover, elderly patients were classified as a special cohort in the guidelines and featured in some specific sections considering their extremely high risk.

Recent studies have shown that the populations of elderly patients are increasing with the increasing human life expectancy. Elderly patients always have more complex clinical conditions and worse prognoses than younger patients (1). Therefore, the effect of staged PCI on MVD in such patients is uncertain. However, the most randomized controlled trials (RCTs) exclude elderly patients. No study has aimed to identify the preferred strategy for these patients. Therefore, this study conducted a comprehensive analysis to compare the effects of staged PCI vs. culprit-only PCI on MVD in elderly patients with STEMI to provide ideas for future treatment strategies.

## Materials and methods

### Study design and population

This single-center retrospective observational study received no sponsorship from enterprises. A total of 1,592 patients aged ≥65 years with STEMI who had undergone primary PCI of the culprit vessels within 12 h of symptom onset from January 2014 to September 2019 at Tianjin Chest Hospital were included. MVD was defined as angiographic stenosis ≥70% in ≥2 major coronary arteries (diameter ≥ 2.5 mm). Patients were excluded if they met the following criteria: (1) single-vessel disease (*n* = 622); (2) planned coronary artery bypass graft (CABG) surgery after primary PCI (*n* = 209); (3) immediate complete revascularization and staged PCI performed >90 days after primary PCI (*n* = 36); (4) stenosis of the left main coronary artery ≥50% or concomitant chronic occlusion disease (*n* = 56); (5) cardiac shock or death before hospital discharge (*n* = 7); (6) previous CABG (*n* = 3); and (7) lack of complete clinical data (*n* = 20). A total of 639 patients were remained; of them, 617 patients for whom full clinical data with follow-up information were available were included in the final analyses. The research protocol was authorized by the Tianjin Chest Hospital Ethical Committee and conducted in accordance with the Declaration of Helsinki. This was a retrospective cohort study from real-world situations; therefore, written informed consent from the enrolled patients was not required. The authors vouch for the completeness and accuracy of the data used in this study.

### Study procedures

All primary PCI procedures were performed according to current guidelines. The culprit vessel was identified based on electrocardiographic changes and echocardiographic and angiographic findings. According to the treatment strategies, the patients were further categorized into the culprit-only PCI group (only culprit vessels were revascularized by primary PCI) and staged PCI groups (non-culprit vessels were staged revascularized after primary PCI). Non-culprit vessels of patients in the staged PCI group were revascularized within 90 days (12) after the primary PCI. All baseline data were obtained from the patients’ medical records by two independent investigators who were blinded to the study parameters. Baseline information included demographics, clinical characteristics, laboratory data, angiographic and procedural details, and medication at discharge.

### Follow-up and endpoints

All patients were followed up from December 2021 to January 2022 by telephone or outpatient visits. The primary endpoint was major adverse cardiac and cerebrovascular events (MACCE), defined as a composite of all-cause death, recurrent myocardial infarction (MI), stroke, and ischemia-driven revascularization. The secondary endpoints included all-cause death, cardiac death, recurrent MI, stroke, and ischemia-driven revascularization. All-cause death was defined as death of any cause. Cardiac death was defined as death caused by cardiovascular disease, such as acute MI, heart failure, and cardiac arrhythmia.

### Statistical analysis

Continuous variables were expressed as mean ± standard deviation (SD) if normally distributed or as median and interquartile range if non-normally distributed. These were compared using the *t*-test or the Mann-Whitney *U*-test. Categorical variables were presented as frequency or percentage and were contrasted using the chi-squared test or Fisher’s exact test. Survival analyses were conducted using Kaplan-Meier curves and contrasted using the log-rank test. Multivariate Cox proportional hazard regression analysis with an entry/exit criterion of 0.1/0.1 was used to acquire the hazard ratios (HRs) and 95% confidence intervals (CIs) to identify independent predictors of MACCE and all-cause death. All variables in Tables 1, 2, except total stent length, are input into the univariate model and then input into a multivariate model with *p* < 0.10. Before propensity score matching (PSM), possible factors of MACCE included staged PCI, diabetes, previous stroke, estimated glomerular filtration rate (eGFR), heart rate, LVEF, Killip class, use of beta-blocker, intra-aortic balloon pump (IABP) use, number of narrowed coronary arteries, non-culprit lesion diameter stenosis, and use of drug-eluting stents. Possible factors of all-cause death included staged PCI, age, hypertension, diabetes, atrial fibrillation, previous stroke, previous smoking, heart rate, LVEF, eGFR, peak troponin level, Killip class, use of beta-blockers, IABP, culprit vessel, number of narrowed coronary arteries, non-culprit lesion diameter stenosis, and use of drug-eluting stents. After PSM, the possible factors of MACCE included staged PCI, previous stroke, peak creatine kinase myocardial band (CK-MB) level, culprit vessel, and the number of narrowed coronary arteries. Possible factors for all-cause death included staged PCI, previous stroke, LVEF, and eGFR. To adjust for confounding factors, differences in clinical outcomes between the two groups were compared using PSM with a matching ratio of 1:1 and caliper value of 0.02. The matching variables included all baseline, angiographic, and procedural characteristics, as well as medication at discharge. All analyses were performed using SPSS version 25.0 (IBM Corp., Armonk, NY, United States). Two-tailed tests were considered statistically significant at a level of 0.05.

**TABLE 1:** Baseline characteristics of included patients before and after propensity score matching (PSM).

Characteristic	Before PSM analysis			After PSM analysis		
	Staged PCI (*n* = 154)	Culprit-only PCI (*n* = 463)	*P*-value	Staged PCI (*n* = 80)	Culprit-only PCI (*n* = 80)	*P*-value
Age, years	70.6 ± 4.7	73.8 ± 6.5	<0.001	68.7 ± 2.8	68.7 ± 2.8	0.772
Male, no. (%)	105 (68.2)	267 (57.7)	0.021	51 (63.8)	54 (67.5)	0.736
Hypertension, no. (%)	107 (69.5)	318 (68.7)	0.853	53 (66.3)	52 (65.0)	1.000
Diabetes, no. (%)	51 (33.1)	149 (32.2)	0.830	23 (28.8)	24 (30.0)	1.000
Dyslipidemia, no. (%)	93 (60.4)	285 (61.6)	0.311	49 (61.3)	49 (61.3)	1.000
Atrial fibrillation, no. (%)	5 (3.2)	22 (4.8)	0.429	4 (5.0)	3 (3.8)	1.000
Previous stroke, no. (%)	29 (18.8)	128 (27.6)	0.030	20 (25.0)	23 (28.8)	0.711
Previous MI, no. (%)	8 (5.2)	27 (5.8)	0.767	4 (5.0)	8 (10.0)	0.388
Previous PCI, no. (%)	12 (7.8)	55 (11.9)	0.158	6 (7.5)	11 (13.8)	0.332
Previous smoker, no. (%)	26 (16.9)	59 (12.7)	0.197	11 (13.8)	13 (16.3)	0.804
Current smoker, no. (%)	59 (38.3)	168 (36.3)	0.651	31 (38.8)	29 (36.3)	0.878
Systolic blood pressure, mmHg	128.7 ± 24.5	131.1 ± 26.1	0.298	128.9 ± 23.7	124.2 ± 26.4	0.265
Heart rate, beats/min	68.0 ± 13.0	70.8 ± 16.8	0.035	68.8 ± 13.0	67.0 ± 13.8	0.406
LVEF,%	52 (46−57)	49 (43−55)	<0.001	51.5 (46.0−56.0)	50.0 (44.0−55.0)	0.252
Triglycerides, mmol/L	1.4 (1.0−1.9)	1.2 (0.9−1.7)	0.044	1.4 (1.0−1.8)	1.3 (1.0−1.8)	0.685
Total cholesterol, mmol/L	4.6 ± 1.0	4.5 ± 1.0	0.663	4.6 ± 1.1	4.7 ± 1.0	0.371
HDL-C, mmol/L	1.1 ± 0.2	1.1 ± 0.4	0.002	1.1 ± 0.2	1.1 ± 0.3	0.699
LDL-C, mmol/L	3.1 ± 0.9	3.1 ± 0.9	0.299	3.1 ± 1.0	3.2 ± 0.8	0.782
eGFR, mL/min/1.73 m^2^	79.0 ± 19.7	74.2 ± 23.3	0.012	80.5 ± 19.9	81.5 ± 20.4	0.716
Peak troponin, ng/mL	3.7 (1.4−7.7)	4.2 (1.4−9.0)	0.354	3.7 (1.1−8.4)	4.3 (1.4−8.2)	0.705
Peak CK-MB, U/L	114 (53.8−201.3)	142 (65.8−255.0)	0.053	112.5 (58.3−211.0)	132.0 (66.3−246.3)	0.379
Killip class, no. (%)			0.059			0.919
1	145 (94.2)	399 (86.2)		75 (93.8)	73 (91.3)	
2	6 (3.9)	46 (9.9)		3 (3.8)	5 (6.3)	
3	1 (0.6)	8 (1.7)		1 (1.3)	1 (1.3)	
4	2 (1.3)	10 (2.2)		1 (1.3)	1 (1.3)	
Time from symptom onset to primary PCI, h	4 (2−6)	4 (2−6)	0.991	3.5 (2.0−5.8)	3.3 (2.0−6.0)	0.826
**Medications at discharge, no. (%)**						
Aspirin	152 (98.7)	453 (97.8)	0.503	79 (98.8)	80 (100.0)	1.000
Clopidogrel	123 (79.9)	384 (82.9)	0.389	64 (80.0)	64 (80.0)	1.000
Ticagrelor	31 (20.1)	74 (16.0)	0.235	16 (20.0)	16 (20.0)	1.000
Beta-blocker	121 (78.6)	365 (78.8)	0.945	63 (78.8)	64 (80.0)	1.000
ACEI/ARB	121 (78.6)	345 (74.5)	0.310	63 (78.8)	59 (73.8)	0.597
Statins	148 (96.1)	441 (95.2)	0.659	77 (96.3)	76 (95.0)	1.000

Data are expressed as percentages, mean ± standard deviation (SD) or median and interquartile range. The p-Values represent differences in baseline characteristics between the two groups. PSM, propensity score matching; PCI, percutaneous coronary intervention; MI, myocardial infarction; LVEF, left ventricular ejection fractions; HDL-C, high-density lipoprotein-cholesterol; LDL-C, low-density lipoprotein-cholesterol; eGFR, estimated glomerular filtration rate; CK-MB, creatine kinase myocardial band; ACEI, angiotensin-converting enzyme inhibitor; ARB, angiotensin receptor blocker.

**TABLE 2:** Angiographic characteristics and procedural characteristics of included patients before and after PSM.

Characteristic	Before PSM analysis			After PSM analysis		
	Staged PCI (*n* = 154)	Culprit-only PCI (*n* = 463)	*P*-value	Staged PCI (*n* = 80)	Culprit-only PCI (*n* = 80)	*P*-value
Thrombus aspiration, no. (%)	56 (36.4)	180 (38.9)	0.578	21 (26.3)	33 (41.3)	0.081
IABP, no. (%)	2 (1.3)	47 (10.2)	<0.001	1 (1.3)	3 (3.8)	0.625
Culprit vessel, no./total culprit vessels (%)			0.007			0.489
Left anterior descending	39/156 (25.0)	179/470 (38.1)		22 (27.5)	24 (30.0)	
Left circumflex	24/156 (15.4)	48/470 (10.2)		11 (13.8)	14 (17.5)	
Right	93/156 (59.6)	243/470 (51.7)		47 (58.8)	42 (52.5)	
No. of narrowed coronary arteries			0.582			0.871
2	96 (62.3)	300 (64.8)		48 (60.0)	50 (62.5)	
3	58 (37.7)	163 (35.2)		32 (40.0)	30 (37.5)	
Non-culprit vessel, no./total non-culprit vessels (%)			0.019			0.657
Left anterior descending	94/212 (44.3)	210/625 (33.6)		42/112 (36.8)	47/110 (42.7)	
Left circumflex	79/212 (37.3)	281/625 (45.0)		46/112 (41.1)	39/110 (35.5)	
Right coronary artery	39/212 (18.4)	134/625 (21.4)		24/112 (21.4)	24/110 (21.8)	
Non-culprit vessel diameter stenosis, no./total non-culprit vessels (%)			<0.001			0.012
70−79%	3/212 (1.4)	43/625 (6.9)		1/112 (0.9)	5/110 (4.5)	
80−89%	54/212 (25.5)	202/625 (32.3)		34/112 (30.4)	36/110 (32.7)	
90−99%	136/212 (64.2)	275/625 (44.0)		68/112 (60.7)	48/110 (43.6)	
100%	19/212 (9.0)	105/625 (16.8)		9/112 (8.0)	21/110 (19.1)	
Type of PCI, no. (%)			0.002			1.000
Drug-eluting stent	152 (98.7)	424 (91.6)		78 (97.5)	79 (98.8)	
PTCA	2 (1.3)	39 (8.4)		2 (2.5)	1 (1.3)	
PCI access, no. (%)			0.348			0.359
Femoral artery	124 (80.5)	388 (83.8)		67 (83.8)	72 (90.0)	
Radial artery	30 (19.5)	75 (16.2)		13 (16.3)	8 (10.0)	
Minimum stent diameter, mm	2.8 (2.5−3.0)	3.0 (2.8−3.5)	<0.001	2.8 (2.5−3.0)	2.8 (2.5−3.0)	0.124
Total stent length, mm	74.4 ± 27.0	37.7 ± 19.4	<0.001	72.4 ± 28.0	40.2 ± 21.8	<0.001

PSM, propensity score matching; PCI, percutaneous coronary intervention; IABP, intra-aortic balloon pump; PTCA, percutaneous transluminal coronary angioplasty.

## Results

### Baseline, angiographic, and procedural characteristics

Among the 617 elderly patients with STEMI and MVD who underwent primary PCI of the culprit vessels, 463 (75%) underwent culprit-only PCI and 154 (25%) underwent staged PCI.

After PSM, 160 patients were matched. The mean follow-up period was 56 months (interquartile range, 40–89 months). The baseline information and laboratory, angiographic, and procedural characteristics of the overall patients and the PSM cohort are listed in Tables 1, 2.

Before PSM, patients from the staged PCI group were more likely to be male (*p* = 0.021) and younger (*p* < 0.001); have a history of previous stroke (*p* = 0.030); have a higher LVEF (*p* < 0.001), triglyceride level (*p* = 0.044), and eGFR (*p* = 0.012); and have a lower heart rate (*p* = 0.035) and high-density lipoprotein cholesterol (HDL-C) level (*p* = 0.002) than those in the culprit-only PCI group. Moreover, patients who underwent staged PCI were less likely to receive IABP (*p* < 0.001), more likely to receive drug-eluting stents (*p* = 0.002), more likely to have non-culprit vessels including the left circumflex artery and right coronary artery (*p* = 0.019), and less likely to have culprit vessels including the left anterior descending coronary artery (*p* = 0.007). What is more, they had a shorter minimum stent diameter and total stent length (both *p* < 0.001). Non-culprit vessel diameter stenosis in the staged PCI group was prone to be 90–99%, while that in the culprit-only PCI group was prone to be 70–79%, 80–89%, and 100% (*p* < 0.001). The other characteristics did not differ significantly between groups.

After PSM, baseline information and laboratory, angiographic, and procedural characteristics were fully consistent between groups except for non-culprit lesion diameter stenosis (*p* = 0.012; Tables 1, 2).

### Clinical outcomes

During the follow-up period of 56 months, 209 patients had developed MACCE and 119 died.

**FIGURE 1:**
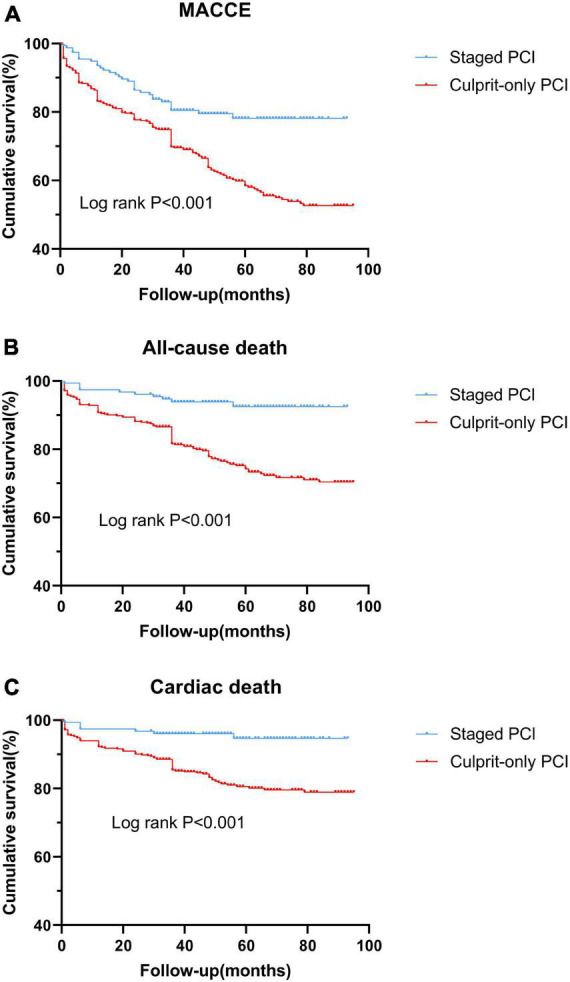
Kaplan-Meier curves of clinical outcomes in the two groups before the propensity score matching analysis. **(A)** MACCE; **(B)** all-cause death; and **(C)** cardiac death. MACCE, major adverse cardiac and cerebrovascular event; PCI, percutaneous coronary intervention.

Before PSM, staged PCI had a lower risk of MACCE (HR, 0.487; 95% CI, 0.332–0.713; *p* < 0.001), all-cause death (HR, 0.264; 95% CI, 0.138–0.504; *p* < 0.001), cardiac death (HR, 0.247; 95% CI, 0.114–0.534; *p* < 0.001), and recurrent MI (HR, 0.318; 95% CI, 0.126–0.799; *p* = 0.015) than culprit-only PCI (Figure 1 and Table 3). Other clinical outcomes, such as stroke and ischemia-driven revascularization, did not differ significantly between groups. Cox proportional hazard regression revealed that the staged PCI group had a reduced risk of MACCE (HR, 0.579; 95% CI, 0.390–0.860; *p* = 0.007) and all-cause death (HR, 0.420; 95% CI, 0.212–0.832; *p* = 0.013) in contrast with culprit-only PCI (Tables 4, 5). Staged PCI played a robust role in predicting the risk of a lower incidence of MACCE and all-cause death. Furthermore, previous stroke (*p* = 0.006) and Killip class (*p* = 0.015) were independent predictors of MACCE (Table 4). Age (*p* = 0.006), previous smoking status (*p* = 0.044), eGFR (*p* = 0.003), and Killip class (*p* = 0.014) were also independent predictors of all-cause mortality (Table 5).

**TABLE 3:** Clinical outcomes between the two groups before and after the PSM analysis.

Outcome	Before PSM analysis				After PSM analysis			
	Staged PCI (*n* = 154)	Culprit-only PCI (*n* = 463)	HR (95% CI)	*P*-value	Staged PCI (*n* = 80)	Culprit-only PCI (*n* = 80)	HR (95% CI)	*P*-value
MACCE	31 (20.1)	178 (38.4)	0.487 (0.332−0.713)	<0.001	15 (18.8)	31 (38.8)	0.468 (0.252−0.867)	0.016
All-cause death	10 (6.5)	109 (23.5)	0.264 (0.138−0.504)	<0.001	3 (3.8)	15 (18.8)	0.185 (0.054−0.636)	0.007
Cardiac death	7 (4.5)	82 (17.7)	0.247 (0.114−0.534)	<0.001	1 (1.3)	13 (16.3)	0.075 (0.010−0.577)	0.013
Recurrent MI	5 (3.2)	47 (10.2)	0.318 (0.126−0.799)	0.015	3 (3.8)	9 (11.3)	0.347 (0.094−1.281)	0.112
Stroke	6 (3.9)	20 (4.3)	0.953 (0.382−2.379)	0.918	4 (5.0)	2 (2.5)	2.192 (0.400−12.021)	0.366
Ischemia-driven revascularization	16 (10.4)	57 (12.3)	0.868 (0.498−1.513)	0.619	9 (11.3)	13 (16.3)	0.735 (0.314−1.720)	0.478

PSM, propensity score matching; PCI, percutaneous coronary intervention; HR, hazard ratio; CI, confidence interval; MACCE, major adverse cardiac and cerebrovascular events; MI, myocardial infarction.

**TABLE 4:** Cox proportion hazards analysis for predictors of major adverse cardiac and cerebrovascular events (MACCE) before PSM analysis.

Variable	Univariate analysis		Multivariate analysis	
	HR (95% Cl)	*P*-value	HR (95% Cl)	*P*-value
Staged PCI (vs. culprit-only PCI)	0.487 (0.332−0.713)	<0.001	0.579 (0.390−0.860)	0.007
Diabetes	1.487 (1.127−1.961)	0.005	1.243 (0.928−1.664)	0.145
Previous stroke	1.822 (1.370−2.422)	<0.001	1.531 (1.133−2.068)	0.006
eGFR, mL/min/1.73 m^2^	0.994 (0.987−1.000)	0.045	0.997 (0.991−1.004)	0.397
Heart rate, beats/min	1.015 (1.006−1.023)	<0.001	1.007 (0.999−1.016)	0.102
LVEF, %	0.981 (0.964−0.998)	0.026	0.998 (0.979−1.017)	0.833
Killip class	1.411 (1.171−1.701)	<0.001	1.285 (1.049−1.574)	0.015
Use of beta-blocker	1.391 (0.967−2.000)	0.075	1.210 (0.830−1.764)	0.323
IABP	1.929 (1.279−2.910)	0.002	1.1094 (0.755−1.889)	0.449
No. of narrowed coronary arteries	1.259 (0.982−1.709)	0.067	1.163 (0.856−1.581)	0.333
Non-culprit lesion diameter stenosis	1.197 (0.999−1.433)	0.051	1.126 (0.927−1.368)	0.231
Use of drug-eluting stent	0.657 (0.410−1.053)	0.081	0.820 (0.502−1.341)	0.429

HR, hazard ratio; CI, confidence interval; PCI, percutaneous coronary intervention; eGFR, estimated glomerular filtration rate; LVEF, left ventricular ejection fraction; IABP, intra-aortic balloon pump.

**TABLE 5:** Cox proportion hazards analysis for predictors of all-cause death before PSM analysis.

Variable	Univariate analysis		Multivariate analysis	
	HR (95% Cl)	*P*-value	HR (95% Cl)	*P*-value
Staged PCI (vs. culprit-only PCI)	0.264 (0.138−0.504)	<0.001	0.420 (0.212−0.832)	0.013
Age	1.069 (1.041−1.097)	<0.001	1.043 (1.012−1.075)	0.006
Hypertension	1.422 (0.940−2.151)	0.096	1.104 (0.713−1.710)	0.656
Diabetes	1.653 (1.149−2.376)	0.007	1.352 (0.900−2.028)	0.146
Atrial fibrillation	2.119 (1.018−4.051)	0.023	1.182 (0.586−2.386)	0.641
Previous stroke	1.928 (1.329−2.799)	<0.001	1.460 (0.978−2.179)	0.064
Previous smoker	1.609 (1.028−2.518)	0.037	1.605 (1.012−2.547)	0.044
Heart rate, beats/min	1.027 (1.017−1.037)	<0.001	1.010 (0.999−1.021)	0.069
LVEF, %	0.949 (0.929−0.970)	<0.001	0.978 (0.952−1.006)	0.117
eGFR, mL/min/1.73 m^2^	0.976 (0.968−0.984)	<0.001	0.987 (0.978−0.996)	0.003
Peak troponin, μmol/L	1.052 (1.001−1.106)	0.047	0.990 (0.939−1.044)	0.715
Killip class	1.703 (1.388−2.088)	<0.001	1.357 (1.063−1.733)	0.014
Use of beta-blocker	1.552 (0.940−2.562)	0.086	1.127 (0.664−1.912)	0.658
IABP	3.780 (2.431−5.878)	<0.001	1.502 (0.886−2.547)	0.110
Culprit vessel	0.719 (0.538−0.960)	0.025	1.048 (0.739−1.485)	0.792
No. of narrowed coronary arteries	1.393 (0.968−2.005)	0.074	1.087 (0.719−1.642)	0.694
Non-culprit lesion diameter stenosis	1.358 (1.067−1.728)	0.013	1.202 (0.932−1.550)	0.156
Use of drug-eluting stent	0.530 (0.298−0.944)	0.031	0.842 (0.449−1.576)	0.590

HR, hazard ratio; CI, confidence interval; PCI, percutaneous coronary intervention; LVEF, left ventricular ejection fractions; eGFR, estimated glomerular filtration rate; IABP, intra-aortic balloon pump.

**FIGURE 2:**
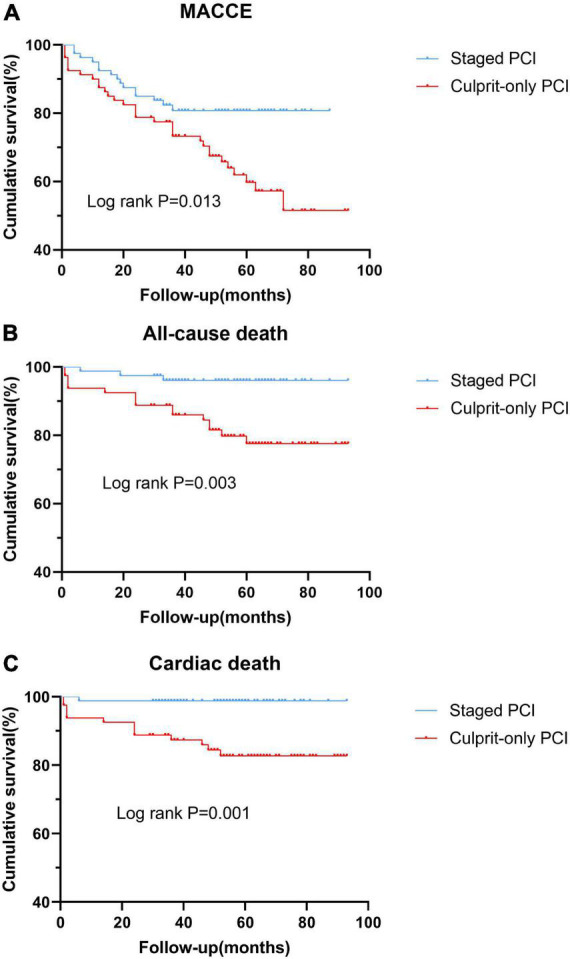
Kaplan-Meier curves of clinical outcomes in the two groups after the propensity score matching analysis. **(A)** MACCE; **(B)** all-cause death; and **(C)** cardiac death. MACCE, major adverse cardiac and cerebrovascular event; PCI, percutaneous coronary intervention.

After PSM, the staged PCI group still had a lower risk of MACCE (HR, 0.468; 95% CI, 0.252–0.867; *p* = 0.016), all-cause death (HR, 0.185; 95% CI, 0.054–0.636; *p* = 0.007), and cardiac death (HR, 0.075; 95% CI, 0.010–0.577; *p* = 0.013) than the culprit-only group (Figure 2 and Table 3). Other clinical outcomes rarely showed significant differences, such as recurrent MI, stroke, and ischemia-driven revascularization. In addition, Cox proportional hazard regression analysis indicated that staged PCI reduced the risk of MACCE (HR, 0.499; 95% CI, 0.267–0.932; *p* = 0.029) and all-cause death (HR, 0.192; 95% CI, 0.056–0.660; *p* = 0.009; Tables 6, 7). After PSM, the role of staged PCI in predicting the risk of MACCE and all-cause mortality remained robust. Moreover, the previous stroke was an independent predictor of MACCE (*p* = 0.019) and all-cause death (*p* = 0.009; Tables 6, 7).

**TABLE 6:** Cox proportion hazards analysis for predictors of the MACCE after PSM analysis.

Variable	Univariate analysis		Multivariate analysis	
	HR (95% Cl)	*P*-value	HR (95% Cl)	*P*-value
Staged PCI (vs. culprit-only PCI)	0.468 (0.252−0.867)	0.016	0.499 (0.267−0.932)	0.029
Previous stroke	2.477 (1.380−4.448)	0.002	2.076 (1.126−3.829)	0.019
Peak CK-MB, U/L	1.001 (1.000−1.002)	0.082	1.001 (1.000−1.002)	0.168
Culprit vessel	1.491 (0.955−2.327)	0.079	1.388 (0.887−2.173)	0.151
No. of narrowed coronary arteries	2.099 (1.175−3.752)	0.012	1.729 (0.935−3.195)	0.081

HR, hazard ratio; CI, confidence interval; PCI, percutaneous coronary intervention; CK-MB, creatine kinase myocardial band.

**TABLE 7:** Cox proportion hazards analysis for predictors of all-cause death after PSM analysis.

Variable	Univariate analysis		Multivariate analysis	
	HR (95% Cl)	*P*-value	HR (95% Cl)	*P*-value
Staged PCI (vs. culprit-only PCI)	0.185 (0.054−0.636)	0.007	0.192 (0.056−0.660)	0.009
Previous stroke	3.461 (1.405−8.526)	0.007	3.383 (1.354−8.450)	0.009
LVEF,%	0.942 (0.895−0.991)	0.021	0.948 (0.897−1.001)	0.056
eGFR, mL/min/1.73 m^2^	0.977 (0.956−0.999)	0.041	0.979 (0.957−1.002)	0.071

HR, hazard ratio; CI, confidence interval; PCI, percutaneous coronary intervention; LVEF, left ventricular ejection fractions; eGFR, estimated glomerular filtration rate.

## Discussion

In this study, among patients with STEMI and MVD aged ≥ 65 years, staged PCI showed a lower incidence of MACCE, all-cause death, and cardiac death than culprit-only PCI in the overall patients and PSM cohorts. This confirmed that, even in elderly adults, staged PCI was more beneficial for patients with STEMI and MVD.

ST-segment elevation myocardial infarction and MVD are associated with worse clinical prognoses. The optimal revascularization strategy for these patients was determined several years ago. Culprit-only PCI was recommended based on previous RCTs and observational studies (6–9). Early practice guidelines (13, 14) also unequivocally supported culprit-only PCI as long as patient hemodynamics remained stable. However, over time, several RCTs, such as the Third Danish Study of Optimal Acute Treatment of Patients with ST-segment Elevation Myocardial Infarction (DANAMI3-PRIMULTI) trial (15) and the Complete vs. Lesion-only Primary PCI Trail (CvLPRIT) (16), elucidated the promising effects of complete revascularization (at primary or staged PCI). The 2015 American College of Cardiology (ACC)/American Heart Association (AHA) updated the guidelines for complete revascularization during primary PCI from III to IIb for MVD in patients with STEMI and stable hemodynamics (17). Subsequently, the Complete vs. Culprit-Only Revascularization Strategies to Treat Multivessel Disease after Early PCI for STEMI (COMPLETE) trial (10) showed that staged PCI had better efficacy when compared with culprit-only PCI. Bates et al. (11) confirmed that when significant non-culprit vessels were suitable candidates for PCI, staged procedures had better efficacy than multivessel primary PCI. After that, the latest 2017 European Society of Cardiology (ESC) guidelines upgraded staged PCI to a class IIaA recommendation (1). Staged PCI has become the best treatment strategy for patients with STEMI and MVD. However, its relative benefit in elderly patients with STEMI remains unclear.

As a higher-risk cohort, elderly patients always have lower cardiac reserve, poorer heart function, more comorbidities, higher risk of bleeding, and worse prognosis (1, 18–20), especially those with STEMI and MVD. Coronary arteries in elderly patients tend to complicate endothelial dysfunction, inflammatory reaction, increased vascular fragility, and severe diffused calcification, meaning that unstable plaques may not reside in the culprit vessels only (21, 22). Because of the increased burden of coronary artery disease, they develop the greater potential to benefit from aggressive revascularization treatment (23–26). However, in clinical practice, the greater the risk, the more conservative the strategy, especially in elderly patients (23). Considering the advanced age and complex clinical condition, many elderly patients, their relatives, and doctors often hesitate to choose staged PCI. Their higher tendency to choose culprit-only PCI led to delayed or absent revascularization for non-culprit vessels and may cause high mortality and recurrent MI rates. However, elderly patients were excluded from most RCTs of MVD in patients with STEMI (27). The efficacy of staged revascularization in these patients is uncertain.

The present study indicated that staged PCI significantly reduced the risk of MACCE (HR, 0.487; 95% CI, 0.332–0.713; *p* < 0.001) as compared to culprit-only PCI for MVD in elderly patients with STEMI, primarily due to the reduction in all-cause death (HR, 0.264; 95% CI, 0.138–0.504; *p* < 0.001) and cardiac death (HR, 0.247; 95% CI, 0.114–0.534; *p* < 0.001). This was obviously due to the ability of staged PCI to reduce the ischemic burden of non-culprit vessels and stabilize vulnerable plaques. In addition, staged PCI can increase blood flow to the watershed areas of infarction to improve myocardial salvage and resolve early myocardial stunning/hibernation. It can also improve collateral circulation, reduce MI areas, and slow the deterioration of heart function, especially in elderly patients (16). This may be the reason why staged PCI reduced the mortality of elderly patients with STEMI and MVD. These outcomes were consistent with subgroup analyses of the CvLPRIT, which suggested the benefit of complete revascularization during index admission for patients older than 65 years (16).

A meta-analysis also revealed that complete revascularization decreased ischemic events in patients with MVD at 63 ± 7 years of age (28). Moreover, the present study found that staged PCI decreased the risk of recurrent MI (before PSM: HR, 0.318; 95% CI, 0.126–0.799; *p* = 0.015; after PSM: HR, 0.347; 95% CI, 0.094–1.281; *p* = 0.112). The high rate of recurrence of MI in elderly patients is the result of plaque rupture of non-culprit vessels and may be associated with their more extensive atherosclerosis (29). Delayed or absent complete revascularization may cause patients to miss timely reperfusion of non-culprit vessels and lead to high recurrent MI rates. This may also be the reason that, compared with culprit-only PCI, staged revascularization could reduce the prevalence rates of cardiac death. There were also some outcomes that rarely differed between the two groups, including stroke and ischemia-driven revascularization. This is slightly different from the results of previous studies. Iqbal et al. (8) reported that staged PCI was less prone to developing repeat ischemia-driven revascularization (HR, 0.64; 95% CI, 0.46–0.90; *p* = 0.012) than culprit-only PCI for all ages. This difference is possibly due to the fact that vascular and hemodynamic compromise with age is responsible for adverse outcomes in elderly patients, even given the optimal revascularization treatment (24). It was also influenced by the negative attitude of elderly patients toward repeated interventions.

The differences between this and other studies are as follows. Firstly, the subject design and study population differ from the others. To our knowledge, this is the first study to explore the efficacy of staged vs. culprit-only PCI for MVD in elderly patients with STEMI. Secondly, we had a long mean follow-up period of approximately 56 months (interquartile range, 40–89 months). Thirdly, PSM was conducted to eliminate the influence of confounding factors between groups to increase the study’s reliability and feasibility. Our study supports the view that aggressive staged PCI for MVD in elderly patients with STEMI could improve their prognosis and that staged PCI should not be withheld only due to age. These findings will encourage more elderly patients with STEMI and MVD to receive timely staged revascularization treatment. We believe that with the progressive availability of newer-generation stent devices, angiography techniques, and intra-vascular molecular imaging, PCI presents fewer intra- and postoperative risks. This may enable non-culprit vessels to receive more adequate and standardized revascularization to reduce unnecessary mortality. Further large-scale prospective cohort studies are also needed to compare the strategies of drug therapy, multivessel primary PCI, and staged PCI and clarify the optimal timing for staged PCI.

This study had some limitations. Firstly, for many reasons, such as inconvenient transportation or a low tendency to seek medical treatment, the number of patients in this field is low in most hospitals. Although this study included all patients treated in our hospital in recent years, the sample size was still small. Therefore, we did not discuss the optimal timing for staged PCI. In the future, we will conduct this study and examine this topic in greater depth. Secondly, after PSM, there was still a difference in non-culprit lesion diameter stenosis (*p* = 0.012), but it was rarely a significant factor in the multivariate analysis. Thirdly, in this study, the coronary arteries were evaluated based on ≥70% stenosis of two or more major coronary branches (diameter ≥ 2.5 mm). Future trials will address the value of endovascular imaging and fractional flow reserve and so on.

## Conclusion

For MVD in elderly patients with STEMI, staged PCI showed a reduced long-term risk of MACCE, all-cause death, and cardiac death as compared to culprit-only PCI. Moreover, staged PCI was a significant independent predictor of MACCE and all-cause mortality. This study confirmed the benefits of staged PCI for MVD in elderly patients with STEMI, providing a reference for future revascularization strategies for such patients.

## Data availability statement

The original contributions presented in this study are included in the article/supplementary material, further inquiries can be directed to the corresponding author/s.

## Ethics statement

The studies involving human participants were reviewed and approved by the Tianjin Chest Hospital. Written informed consent for participation was not required for this study in accordance with the national legislation and the institutional requirements.

## Author contributions

JL, CW, LW, HC, and YL took part in design of this study. JL, JZ, and YH participated in the clinical data collection. JL, LW, and HS performed the statistical analyses. JL drafted this article. All authors made contributions to the present study and approved the submitted manuscript.
